# Effect of high-power radiofrequency combined with a hypocaloric diet on abdominal subcutaneous and visceral fat: a preliminary randomized pilot study

**DOI:** 10.1007/s10103-026-05059-0

**Published:** 2026-11-04

**Authors:** Sidra Kouser, Jesús Rodríguez Lastra, Carmen Torrejon Rueda

**Affiliations:** 1 Inneo Clinic, Barcelona, Spain; 2 Alfonso X El Sabio University, Villanueva de la Cañada, Spain; 3 Centro Médico Starbene, Barcelona, Spain

**Keywords:** Radiofrequency Therapy, Intra-Abdominal Fat, Subcutaneous Fat, Caloric Restriction, Abdominal Obesity, Energy-Based Device

## Abstract

Visceral and subcutaneous abdominal fat are associated with metabolic and cardiovascular risk. This preliminary randomized pilot study evaluated a high-power radiofrequency energy-based device (EBD) treatment, alone or combined with a hypocaloric dietary intervention, for visceral and subcutaneous abdominal fat reduction. Twenty middle-aged participants were randomly allocated to one of two parallel groups: EBD combined with dietary intervention (7 males, 3 females; mean age 58.2 ± 8.89 years) and EBD alone (5 males, 5 females; mean age 54.4 ± 12.5 years). Both groups received 12 EBD sessions over 6 weeks using a four-channel high-power radiofrequency device. Anthropometric, biochemical, and volumetric abdominal fat measurements were assessed at baseline and post-treatment, with visceral fat additionally evaluated at the 3-month follow-up. Both groups demonstrated reductions in abdominal fat volume. The EBD + diet group showed greater reductions in visceral fat (15.0%) and subcutaneous fat (18.6%) than the EBD-only group, which showed reductions of approximately 9.1% and 9.2%, respectively. Between-group change-score analyses favored the EBD + Diet group for weight and BMI, while differences in visceral and subcutaneous fat did not reach statistical significance. Most biochemical variables showed no clear between-group differences, although exploratory between-group differences were observed for triglycerides, insulin, and HOMA-IR. At the 3-month follow-up, mean visceral fat remained below baseline in both groups. Reductions in visceral and subcutaneous abdominal fat were observed in both treated groups. These findings should be interpreted cautiously due to the exploratory design and small sample size. Larger studies are required to confirm efficacy and long-term outcomes.

## Introduction

The accumulation of abdominal fat, both subcutaneous and visceral, is one of the main risk factors for metabolic, cardiovascular, and endocrine diseases. Central obesity is also linked to an increased risk of insulin resistance, type 2 diabetes, and lipid disorders, requiring effective strategies to reduce abdominal obesity. Historically, dietary intervention has been the primary focus in the treatment of abdominal adiposity, and it is widely recognized that a balanced diet substantially aids in the reduction of fat mass and the normalization of metabolic parameters [1, 2]. In clinical practice, a weight loss of 5–10% will generally improve insulin sensitivity and lipid parameters, so the integration of dietary, exercise, and medical follow-up approaches is the most effective in achieving the reduction of abdominal adiposity and its associated risk [3].

Recently, medical technology has evolved considerably, providing new devices to enhance the impact of diet. Among these technologies, high-power radiofrequency has gained attention, particularly with 4-channel energy-delivery systems combined with a belt designed to deliver localized electromagnetic energy to the treated region [4–6]. Previous studies have suggested that radiofrequency-induced thermal effects may promote lipolytic responses and adipocyte apoptosis under appropriate thermal conditions [7]. Controlled thermal energy delivered to adipose tissue may contribute to fat reduction through several cellular and molecular mechanisms. Experimental studies have suggested that exposure of adipose tissue to temperatures of approximately 43–45 °C may activate caspase-mediated adipocyte apoptosis, increase the apoptotic index, and promote subsequent adipose-tissue remodeling and reductions in adipocyte size [8]. Radiofrequency-induced thermal stimulation may also influence inflammatory and metabolic signaling pathways, including NF-κB, TNF-α, IL-6, insulin signaling, and GLUT4 translocation [9]. Preclinical studies have additionally suggested that thermal stimulation may increase UCP1 expression, mitochondrial density, and the conversion of white adipose tissue into metabolically active beige adipose tissue [10]. Furthermore, lipolysis may be promoted through activation of the cyclic adenosine monophosphate/protein kinase A pathway, resulting in phosphorylation of hormone-sensitive lipase and perilipin proteins and facilitating the release of fatty acids and glycerol [7, 11]. As these molecular pathways were not directly measured in the present study, they should be considered potential mechanisms underlying the observed changes in abdominal adiposity.

On the other hand, a healthy diet decreases caloric intake and improves metabolic parameters, promoting the mobilization and elimination of stored fatty acids [2]. Dietary intervention on visceral and subcutaneous abdominal fat is achieved by maintaining a caloric deficit for an extended period, increasing the quality of food and fiber, and consuming a greater amount of plant-based foods. Small randomized trials indicate that dietary programs are effective in providing marked reductions in visceral adipose tissue (VAT) area and liver fat assessed with MRI/CT for 8 to 12 weeks with clinically meaningful differences compared to a usual diet [12, 13]. Previous studies and systematic reviews have suggested that only calorie restriction has a clear effect on reducing VAT, and nutritional protocols that emphasize a high intake of unprocessed and higher-fiber foods typically correlate with better metabolic and body composition outcomes [14, 15].

The main dietary patterns fall into five fundamental categories: the Mediterranean pattern, Dietary Approaches to Stop Hypertension (DASH), low-carb diets, intermittent fasting, and whole-food, plant-based diets. Visceral fat (VF) can be reduced through sustained caloric deficits, but dietary patterns such as the Mediterranean diet and DASH hold special promise in long-term changes in the cardiometabolic system [16].

In this study, a semi-open hypocaloric diet was incorporated with a target macronutrient distribution of 40–50% carbohydrates, 15% proteins, and 30% fats, applied through flexible food groups, photographic portion control, and weekly nutritional counseling, complemented by the prescription of walking for 1 h/day and the elimination of alcohol and high-glycemic load foods. The novelty of the present study lies in the randomized comparison of EBD alone versus EBD combined with a hypocaloric diet, using MRI-based assessment of visceral and subcutaneous abdominal fat. Therefore, this pilot study aimed to evaluate whether combining a semi-open hypocaloric diet with high-power radiofrequency therapy enhances the reduction of visceral and subcutaneous abdominal fat and improves body composition and metabolic control.

## Subjects and methods

### Study design, participants and sample size

This preliminary randomized pilot study with two parallel independent groups was conducted at the INNEO Clinic (Sant Joan Despí) and the Starbene Medical Center, Barcelona, Spain. Participants were recruited between March and May 2024. Participants were recruited through social media advertisements, printed brochures displayed at different locations, and referrals from other clinics and medical centers contacted by the study team. The intervention and post-treatment assessments were conducted between May and August 2024, and 3-month follow-up assessments were completed in November 2024. The study was not prospectively registered in a clinical trial registry. The participating centers had the required clinical infrastructure, trained staff, and facilities to conduct the EBD intervention and follow-up assessments.

Twenty-seven participants were assessed for eligibility. Eligible participants were adults aged 18–70 years with abdominal adiposity, without clinically significant cardiometabolic disease, and with no contraindications to non-invasive radiofrequency treatment. Participants were excluded if they did not meet these criteria, declined to participate, or were unable to comply with the treatment and follow-up schedule. Seven participants were excluded, and 20 middle-aged adults with abdominal adiposity were randomized into two groups: 10 subjects receiving EBD combined with diet and 10 subjects receiving only the EBD protocol. This study was designed as an exploratory randomized pilot study to evaluate feasibility and preliminary safety, estimate the magnitude and variability of the observed effects, and support the planning of a future confirmatory study. Because reliable previous data for the primary outcome in this population were unavailable, no formal a priori power calculation was performed. A feasibility-based sample of 10 participants per group, for a total of 20 participants, was selected considering the available resources, treatment procedures, and follow-up requirements. The findings are therefore presented as descriptive, exploratory, and hypothesis-generating. The variability and effect estimates obtained from this pilot study may be used to calculate the sample size required for a future adequately powered confirmatory trial.

### Randomization and allocation concealment

**Fig. 1:**
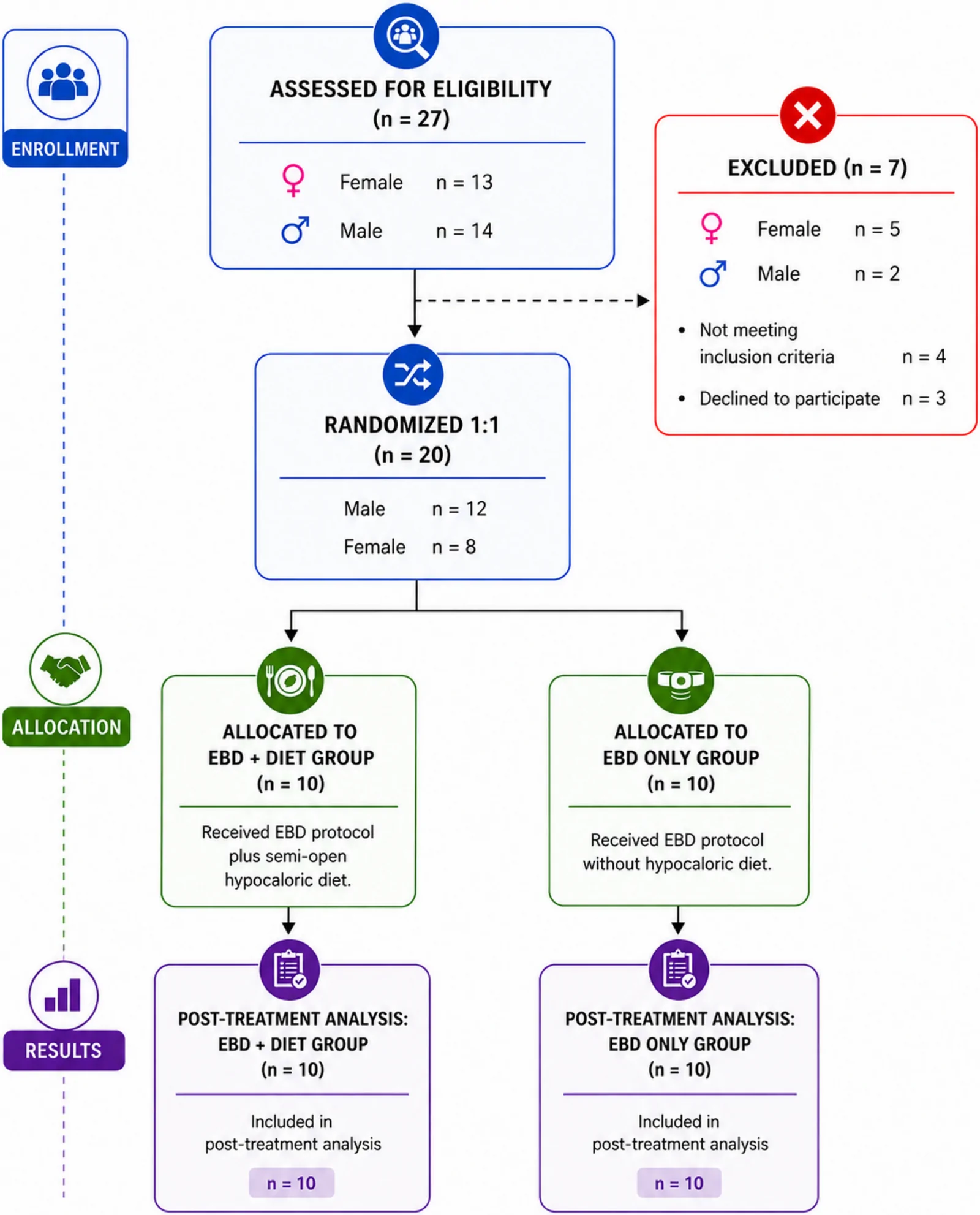
Participant flow diagram showing enrollment, randomization, allocation, follow-up, and analysis

Randomization (1:1) was performed using a computer-generated randomization sequence by an independent investigator who was not involved in subject recruitment, treatment delivery, or subject assessment. Allocations were made using opaque, sealed, and consecutively numbered envelopes stored securely and opened only after eligibility, consent, and baseline evaluations had been confirmed. Allocation concealment was maintained until the envelope was opened. Participant flow is summarized in Fig. 1. All randomized participants attended the planned EBD treatment sessions and completed the post-treatment assessment. In the EBD + Diet group, dietary follow-up was monitored qualitatively through daily meal photographs and weekly counseling.

### Dietary intervention

Participants in the EBD + Diet group followed an individualized semi-open hypocaloric Mediterranean-style diet with a target macronutrient distribution of 40–50% carbohydrates, 15% protein, and 30% fat. Baseline dietary intake was not formally quantified. Estimated basal metabolic rate was calculated using the Mifflin–St Jeor equation: for men, 10 × weight (kg) + 6.25 × height (cm) − 5 × age (years) + 5; and for women, 10 × weight (kg) + 6.25 × height (cm) − 5 × age (years) − 161. Individual caloric recommendations were then set modestly below estimated energy requirements, accounting for usual physical activity. No fixed numerical caloric deficit, expressed as kcal/day or as a percentage, was documented.

The diet was varied and included all major food groups; it was not a dissociated diet, and no complete food group was eliminated. Rice, high-glycemic-index foods, ultra-processed foods, and alcohol were discouraged, although processed meats and higher-fat meat or fish were not strictly prohibited.

Participants were not required to weigh their food. They submitted daily photographs of their meals, which were visually reviewed to monitor portion sizes, food variety, and adherence to the dietary recommendations. The photographs were not used to perform a formal caloric or nutritional calculation. Approximate portion recommendations were adapted according to physical activity level, and participants received weekly nutritional counseling and recommendations to consume approximately 1.5 L of water per day.

All participants were advised to walk for at least 1 h per day. The EBD-only group did not receive dietary counseling or formal dietary monitoring. Consequently, caloric intake could not be compared between the two groups.

### EBD treatment

Both groups received the same EBD protocol using the Drakarian® high-power radiofrequency EBD device (Capenergy Medical, Barcelona, Spain). MRI segmentation was performed by an external imaging provider, and blood biochemical analyses were performed by an external accredited laboratory. The device manufacturer did not perform MRI segmentation or laboratory analyses.

Participants were placed in a supine position. Four emitting electro-terminals (200 cm2 each) were positioned anteriorly over the abdomen using an adjustable C-Belt, with two electro-terminals placed on each side of the abdominal midline, while the corresponding receiving electro-terminals were positioned posteriorly over the lumbar/back region. The emitting electro-terminals were used within the C-Belt gel sleeve to ensure adequate contact and coupling with the body surface.

The device delivered continuous sinusoidal radiofrequency energy and allowed selection among three operating frequencies: 0.8, 1.0, and 1.2 MHz. For each channel, the operating frequency was selected according to the coupling/absorption response displayed by the device, using the frequency that provided the highest energy absorption.

Each treatment session lasted 60 min and was delivered at 310 W/channel across four channels, corresponding to a total nominal power of 1240 W. Treatment intensity could be adjusted during the session according to the coupling response and participant feedback. The device recorded absorbed energy in kilojoules (kJ); across the study cohort, mean absorbed energy was 174.7 ± 15.6 kJ per recorded session.

Temperature was monitored throughout treatment using sensors integrated into the emitting electro-terminals and maintained within the predefined operating range of 45–50 °C under continuous operator observation. During each treatment session, participants were directly asked about discomfort, pain, irritation, or other unusual sensations, and the treated area was clinically observed throughout the procedure. Treatment intensity could be reduced or adjusted if necessary according to participant feedback.

Each participant attended 12 treatment visits over 6 consecutive weeks, with two EBD sessions performed per week. All sessions were conducted by professionals trained according to standardized procedures for preparation, positioning, energy delivery, and monitoring.

Before initiation of the study, the device underwent technical performance verification by a qualified manufacturer technician using appropriate electrical test equipment to confirm correct device operation, including power output. A further technical verification was performed after completion of the treatment period.

No additional concomitant treatment was prescribed beyond the dietary, walking, hydration, and EBD procedures described above.

### Outcome measures

The primary outcomes were changes in visceral fat and subcutaneous fat measured at baseline, post-treatment, and 3-month follow-up. Abdominal magnetic resonance imaging (MRI) assessments were performed at CETIR, Ascires Biomedical Group, Barcelona, Spain, using a 3 T General Electric SIGNA Pioneer MRI system with a Body 36/2 matrix receiving coil. MRI reports provided both fat volume in cm3 and estimated fat mass in grams for subcutaneous and visceral fat compartments. The imaging protocol included abdominal T1 acquisitions with 5-mm slices. Segmentation was performed using active contours over the assessed abdominal volume. Fat mass in grams was calculated in the imaging report from MRI-derived volume using an adipose tissue density of 0.9 kg/L. MRI segmentation was performed by an external imaging provider. The same MRI scanner and acquisition protocol were used at all assessment time points.

Secondary anthropometric outcomes included weight, body mass index (BMI), waist circumference, waist-to-hip ratio, and abdominal skinfold thickness. Waist circumference was measured at the umbilical level using a flexible non-stretch measuring tape, with participants standing in a relaxed position. Abdominal skinfold thickness was measured using a Plicometer at an inferolateral abdominal site, approximately 4–5 cm superior to the inguinal crease. The measurement site was adapted to each participant’s anatomy and maintained consistently at baseline, post-treatment, and follow-up assessments. Anthropometric measurements were performed by the same trained operator at baseline, post-treatment, and follow-up assessments.

Secondary biochemical outcomes included glucose, lipid profile, C-reactive protein, insulin, HOMA-IR, free thyroxine (FT4), and leptin. Blood biochemical analyses were performed by an external accredited laboratory. HOMA-IR was calculated as fasting glucose (mg/dL) × fasting insulin (µU/mL)/405. Lipid parameters were obtained from accredited laboratory reports. LDL cholesterol was reported by the laboratory as a calculated value. VLDL cholesterol was extracted from the laboratory report and was interpreted cautiously as a lipid-derived parameter.

### Blinding

Due to the nature of the dietary intervention, participants could not be blinded to group allocation. The clinicians delivering the EBD treatment were not involved in the dietary intervention and were not informed of participants’ dietary allocation. Anthropometric measurements were performed by the same trained operator at baseline, post-treatment, and follow-up assessments. MRI acquisition and segmentation were performed by an external imaging provider using coded participant identifiers only, without information on group allocation. Outcome assessors were blinded to group allocation whenever feasible.

### Ethics approval and consent to participate

The study was approved by the Ethics Committee for Animal and Human Experimentation of the Autonomous University of Barcelona (25 February 2022; ref. 5324). All participants provided written informed consent to participate and for the publication of clinical images. The study was conducted in accordance with the Declaration of Helsinki (Tables 1 and 2).

**Table 1:** Anthropometric and abdominal fat measurements before and after treatment in both groups

Variable	EBD-only Baseline	EBD-only post-treatment	within-group p-value	EBD + Diet Baseline	EBD + Diet post-treatment	within-group p-value	Between-group p-value
Sex	M = 5; F = 5			M = 7; F = 3			
Age (Years)	54.4 ± 12.5			58.2 ± 8.89			
Height (cm)	170.4 ± 6.8			171.3 ± 9.94			
Weight (kg)	86.27 ± 15.6	85.69 ± 14.9	0.045	95.51 ± 9.5	89.62 ± 11.14	0.001	< 0.001
BMI	29.66 ± 4.83	29.45 ± 4.66	0.020	32.57 ± 2.37	30.54 ± 2.7	0.001	< 0.001
Waist-to-hip ratio	0.90 ± 0.07	0.90 ± 0.12	0.080	0.89 ± 0.07	0.90 ± 0.12	0.241	0.261
Skinfold thickness (mm)	26.94 ± 7.52	21.30 ± 7.24	0.001	23.40 ± 9.10	19.35 ± 8.38	0.006	0.351
Waist circumference (cm)	104.95 ± 15.08	99.64 ± 14.64	< 0.001	109.02 ± 9.80	103.60 ± 8.79	0.012	0.957
VF (cm^3^)	4242.3 ± 2657.84	3855.1 ± 2460.3	0.020	4700.24 ± 1719.59	3996.23 ± 1613.20	0.002	0.158
VF (g)	3818 ± 2392.0	3469.6 ± 2214.2	0.020	4230.22 ± 1547.62	3596.61 ± 1451.88	0.002	0.158
SF (cm^3^)	7922.1 ± 3581.3	7193.4 ± 3538.5	0.016	9046.36 ± 2175.91	7367.73 ± 1815.06	0.003	0.060
SF (g)	7129.9 ± 3223.2	6473.9 ± 3184.7	0.016	8141.73 ± 1958.32	6630.90 ± 1633.61	0.003	0.060

EBD: energy-based device; M: Male; F: Female; VF: visceral fat; SF: subcutaneous fat; BMI: body mass index. Data are presented as mean ± standard deviation (SD). Fat mass in grams was calculated in the imaging report from MRI-derived volume using an adipose tissue density of 0.9 kg/L

After adjustment for baseline values, BMI remained significantly lower in the EBD + Diet group than in the EBD-only group (adjusted difference − 1.76 kg/m2, 95% CI − 2.72 to − 0.80; F(1,17) = 15.03, p = 0.001, partial η2 = 0.469). Adjusted between-group differences did not reach statistical significance for visceral fat mass (adjusted difference − 335.55 g, 95% CI − 697.23 to 26.13; F(1,17) = 3.83, p = 0.067, partial η2 = 0.184) or subcutaneous fat mass (adjusted difference − 824.32 g, 95% CI − 1661.65 to 13.01; F(1,17) = 4.31, p = 0.053, partial η2 = 0.202)

**Table 2:** Biochemical parameters before and after treatment

Variable	EBD-only Baseline	EBD-only post-treatment	within-group p-value	EBD + Diet Baseline	EBD + Diet post-treatment	within-group p-value	Between-group p-value
Glucose (mg/dL)	96.6 ± 9.7	100.1 ± 17.3	0.624	102.2 ± 24.4	97.9 ± 16.75	0.493	0.132
Cholesterol (mg/dL)	189.2 ± 42.7	187.3 ± 38.72	0.846	184 ± 28.7	179.7 ± 51.07	0.470	0.900
Triglycerides (mg/dL)	110.3 ± 44.57	120.8 ± 63.9	0.622	121 ± 75.1	100.1 ± 58.5	0.455	0.041
HDL-Cholesterol (mg/dL)	50.8 ± 8.25	50.4 ± 7.36	0.578	50.4 ± 12.9	47.1 ± 6.3	0.642	0.420
LDL-Cholesterol (mg/dL)	117.2 ± 34.4	112.9 ± 32.4	0.880	109.4 ± 28.89	112.7 ± 41.9	0.446	0.684
VLDL-Cholesterol (mg/dL)	22 ± 8.3	24 ± 12.9	0.624	24.2 ± 15.12	19.9 ± 11.77	0.238	0.046
C-Reactive Protein (mg/L)	4.5 ± 6.3	2.89 ± 2.53	0.010	2.54 ± 3.22	1.48 ± 1.48	0.174	0.293
Insulin (μU/mL)	9.29 ± 6.66	12.25 ± 9.10	0.061	11.84 ± 4.47	7.66 ± 4.19	0.001	< 0.001
HOMA-IR	2.26 ± 1.71	3.09 ± 2.36	0.044	2.89 ± 1.25	1.82 ± 1.10	0.002	< 0.001
FT4 (ng/dL)	0.87 ± 0.1	0.85 ± 0.07	0.659	1.15 ± 0.14	1.107 ± 0.09	0.253	0.557
Leptin (ng/mL)	10.95 ± 8.4	7.29 ± 5.82	0.031	7.47 ± 3.31	6.02 ± 5.04	0.318	0.281

HDL: high-density lipoprotein; LDL: low-density lipoprotein; VLDL: very-low-density lipoprotein; HOMA-IR: homeostatic model assessment for insulin resistance; CRP: C-reactive protein; FT4: free thyroxine; EBD: energy-based device. Data are presented as mean ± standard deviation (SD). Statistical significance was set at p < 0.05

Between-group p-values in Tables 1 and 2 compare baseline-to-post-treatment change scores between the EBD-only group and the EBD + Diet group using independent-samples t-tests

**Fig. 2:**
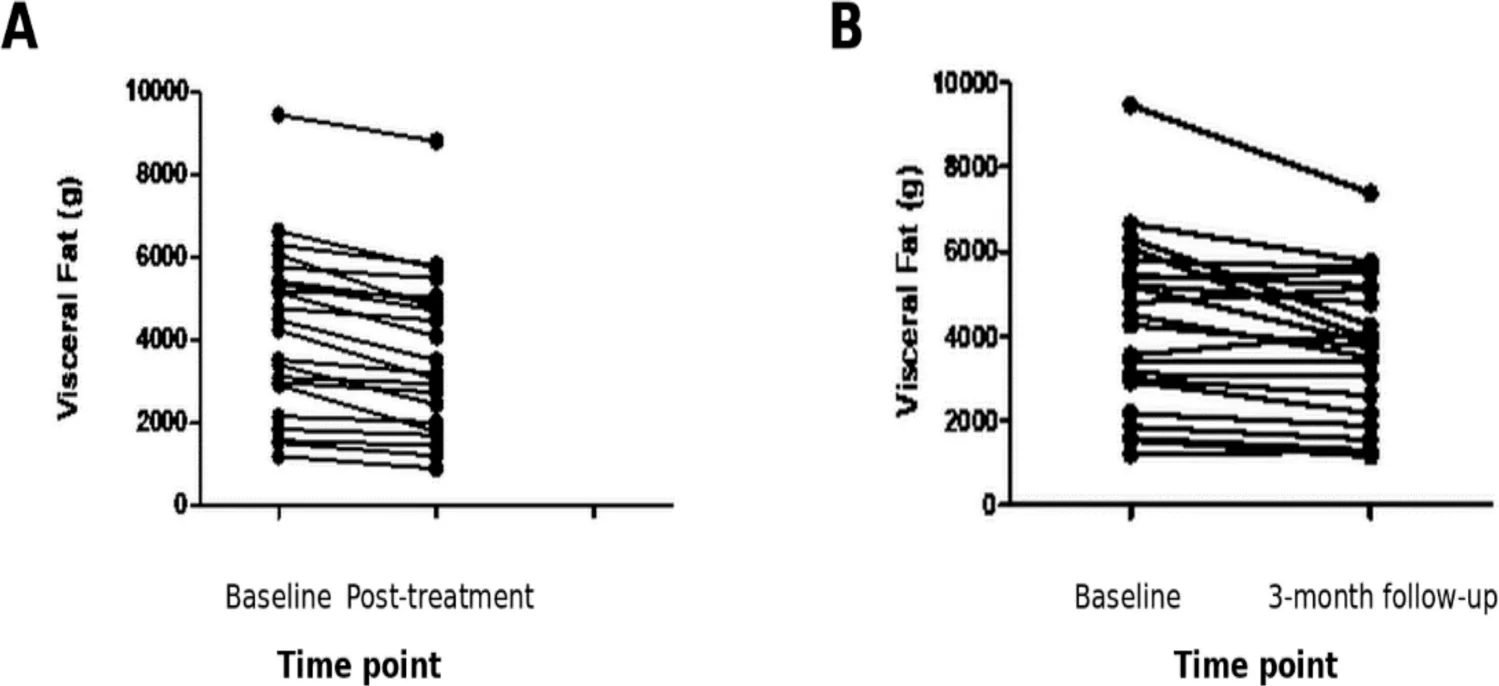
Individual changes in visceral fat from baseline to post-treatment **A** and from baseline to the 3-month follow-up **B**. Each line represents one participant

Three-month follow-up data were available for all participants in the EBD + Diet group (n = 10) and for six participants in the EBD-only group (n = 6). Because Table 3 includes only participants with available 3-month follow-up data, its baseline values were recalculated for this follow-up cohort and therefore differ from the full-cohort baseline values presented in Table 1. At the 3-month follow-up, mean visceral fat remained below baseline in both groups. The reduction from baseline to 3 months was statistically significant in the EBD + Diet group (p = 0.014), whereas the reduction in the EBD-only group did not reach statistical significance (p = 0.093). The corresponding paired effect sizes were large (Cohen’s dz = 0.97 and 0.85, respectively), although these estimates should be interpreted cautiously because of the small follow-up sample. Individual changes in visceral fat values at baseline, post-treatment, and 3-month follow-up are presented in Fig. 2

### Statistical analysis

Continuous variables are presented as mean ± standard deviation (SD). Normality was assessed using the Shapiro–Wilk test, and homogeneity of variances was evaluated using Levene’s test where applicable. Within-group changes between baseline and post-treatment were evaluated using paired-samples t-tests, and between-group differences in baseline-to-post-treatment change scores were assessed using independent-samples t-tests.

For the 3-month follow-up analysis presented in Table 3, within-group changes between baseline and 3-month follow-up were evaluated using paired-samples t-tests. Paired effect sizes were expressed as Cohen’s dz.

**Table 3:** Change in visceral fat mass from baseline to 3-month follow-up by treatment group

Group	n	Baseline mean (g)	3-month Mean ± SD (g)	Mean change ± SD (g)	Percentage change, mean ± SD (%)	p-value	Cohen’s dz
Only EBD	6	4635.18 ± 2834.74	3939.54 ± 2228.89	695.64 ± 823.08	13.42 ± 10.12	0.093	0.85
EBD + Diet	10	4230.22 ± 1547.62	3472.51 ± 1251.38	757.71 ± 782.75	16.89 ± 12.50	0.014	0.97

Percentage change was calculated individually for each participant as [(baseline − 3-month value)/baseline] × 100 and is presented as mean ± SD. Mean change was calculated as baseline minus the 3-month follow-up value. EBD: energy-based device; SD: standard deviation. Data are presented as mean ± SD. Fat mass in grams was calculated in the imaging report from MRI-derived volume using an adipose tissue density of 0.9 kg/L

Between-group post-treatment differences in visceral fat mass, subcutaneous fat mass, and BMI were additionally evaluated using analysis of covariance (ANCOVA), with the post-treatment value as the dependent variable, treatment group as the fixed factor, and the corresponding baseline value as a covariate. Adjusted group differences, 95% confidence intervals (CI), F statistics, p-values, and partial η^2^ were reported.

Given the exploratory nature of the secondary biochemical outcomes and the limited sample size, no formal adjustment for multiple comparisons was applied; therefore, p-values for secondary outcomes were interpreted as nominal and exploratory. Statistical significance was set at p < 0.05. Analyses were considered hypothesis-generating because of the exploratory pilot design.

## Results

Both treatment groups showed reductions in abdominal fat. Compared with the EBD-only group, the EBD + Diet group showed greater reductions in visceral and subcutaneous abdominal fat (Table 1). Waist circumference decreased by 5.31 cm in the EBD-only group and by 5.42 cm in the EBD + Diet group. Visceral and subcutaneous fat decreased by approximately 9.1% and 9.2%, respectively, in the EBD-only group, and by approximately 15.0% and 18.6%, respectively, in the EBD + Diet group.

Between-group comparisons of baseline-to-post-treatment change scores showed statistically significant differences favoring the EBD + Diet group for weight and BMI (both p < 0.001). Between-group differences did not reach statistical significance for waist-to-hip ratio, skinfold thickness, waist circumference, visceral fat (VF cm3 and VF g, p = 0.158), or subcutaneous fat (SF cm3 and SF g, p = 0.060).

For biochemical parameters, between-group differences in change scores were observed for triglycerides (p = 0.041), insulin (p < 0.001), and HOMA-IR (p < 0.001). No statistically significant between-group differences were observed for glucose, total cholesterol, HDL cholesterol, LDL cholesterol, C-reactive protein, FT4, or leptin. Within-group reductions in C-reactive protein and leptin were observed in the EBD-only group. Because of the small sample size, high variability, and absence of multiplicity adjustment, these between-group biochemical findings should be interpreted as nominal and exploratory. Biochemical parameters before and after treatment are presented in Table 2. During treatment, participants reported only a mild warmth sensation. Mild transient erythema was occasionally observed as an expected thermal response. No pain, burns, edema, sensory changes, clinically significant irritation, or other treatment-related adverse effects were reported or clinically observed during the treatment sessions.

### Illustrative clinical and imaging outcomes

**Fig. 3:**
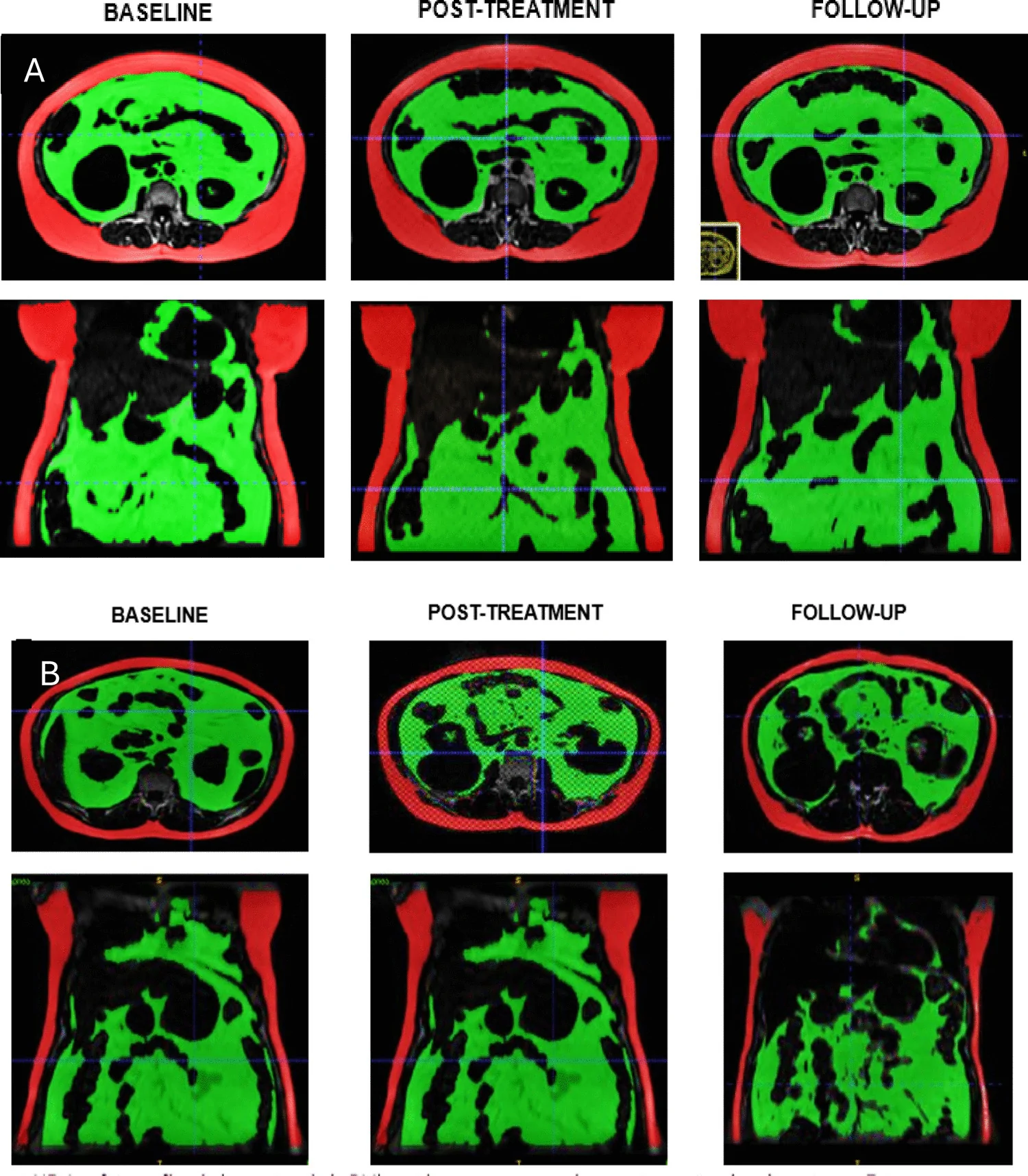
Illustrative MRI evaluations showing abdominal fat distribution at baseline, post-treatment, and 3-month follow-up. (A) A 61-year-old female participant treated with EBD only demonstrated reductions of approximately 20% in subcutaneous fat and 21% in visceral fat after treatment. (B) A 60-year-old male participant treated with EBD combined with dietary intervention demonstrated reductions of approximately 22% in subcutaneous fat and 21% in visceral fat after treatment. These cases were selected to illustrate visible MRI changes after treatment and were not intended to represent the mean response within each group

**Fig. 4:**
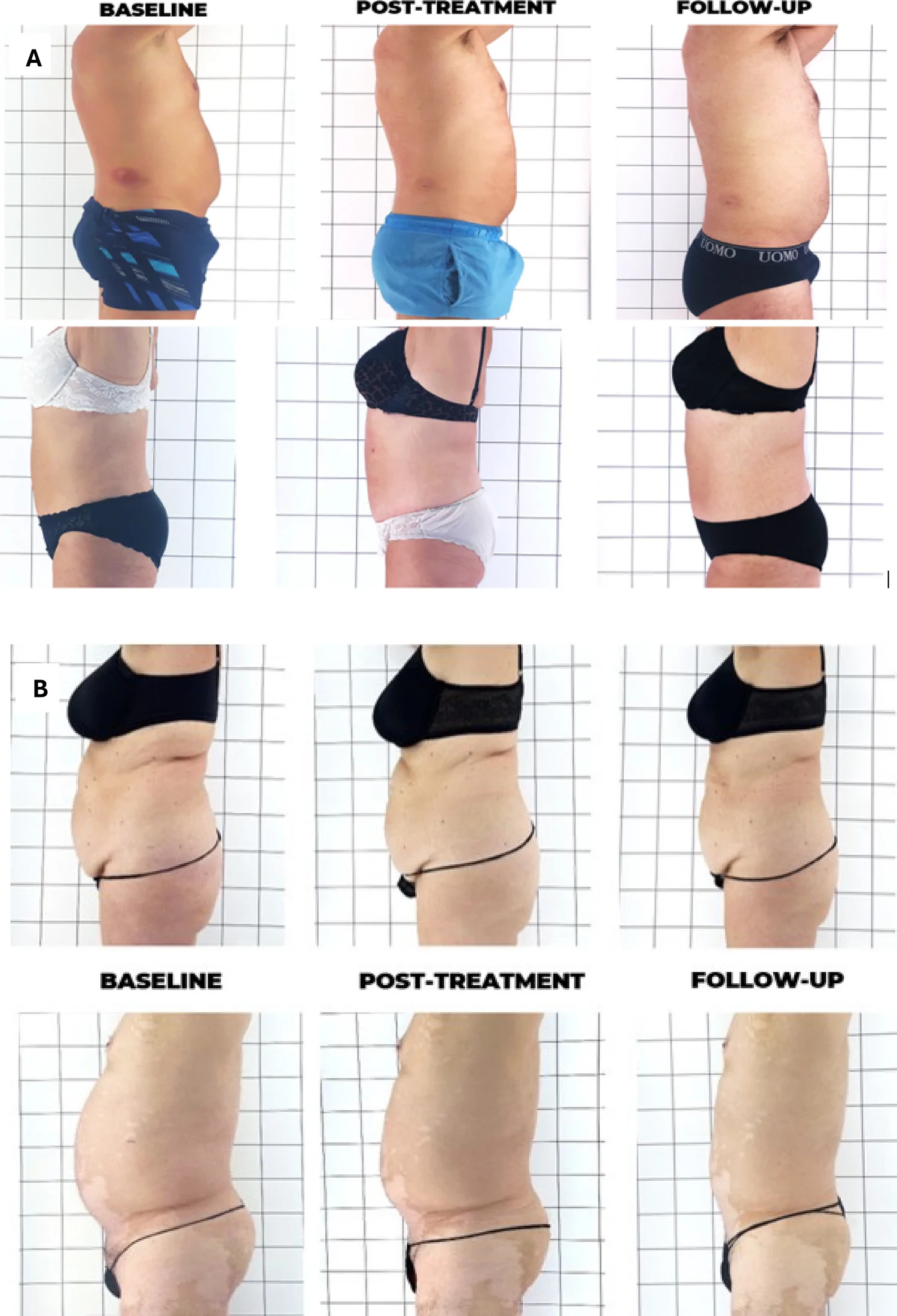
Illustrative clinical photographs showing changes in abdominal volume at baseline, post-treatment, and 3-month follow-up. (A) Participants treated with EBD only. The male participant showed a reduction in waist circumference from 90.4 cm at baseline to 82.1 cm post-treatment and 85.0 cm at follow-up; the female participant showed a reduction from 89.5 cm to 86.6 cm and 85.0 cm, respectively. (B) Participants treated with EBD combined with dietary intervention. The male participant showed a reduction in waist circumference from 109.0 cm at baseline to 103.1 cm post-treatment and 95.5 cm at follow-up; the female participant showed a reduction from 107.7 cm to 105.1 cm and 103.4 cm, respectively

Illustrative MRI and clinical photographic outcomes are shown in Figs. 3 and 4. Individual follow-up trajectories varied, with one EBD-only participant showing an increase in waist circumference from 82.1 cm post-treatment to 85.0 cm at the 3-month follow-up, while remaining below the baseline value of 90.4 cm.

## Discussion

In this comparison of two treated groups, reductions in abdominal fat were observed in both groups, with greater reductions in some outcomes in the EBD + Diet group. The addition of dietary intervention appeared to enhance reductions in body weight, BMI, and subcutaneous fat. The marked reductions in waist circumference despite relatively modest changes in total body weight may reflect regional reductions in visceral and subcutaneous abdominal fat demonstrated by MRI, which are not necessarily proportional to changes in overall body weight. However, because of the small sample size, baseline variability, and exploratory design, these findings should be interpreted cautiously.

Previous studies using CT or MRI to assess visceral fat have also reported significant reductions after dietary interventions. However, most of these studies assessed changes in a single slice, area, or percentage (cm2 or %), whereas the present study used whole-abdomen volumetric fat assessment. When single-slice measurements are converted to estimated fat mass using adipose tissue density assumptions, the resulting values may not correspond to direct whole-abdomen measurements. Therefore, direct comparison with the present results is limited [12–14].

Previous studies reported reductions in subcutaneous fat without modifications in daily habits [5], as well as reductions in visceral and subcutaneous fat when combining treatment with 20 min of submaximal exercise intended to metabolize triglycerides released during lipolysis [6]. The present findings cautiously support the hypothesis that EBD may represent a useful tool for reducing abdominal fat without evidence of inflammatory adverse effects. At the 3-month follow-up, mean visceral fat remained below baseline in both groups, although statistical significance was observed only in the EBD + Diet group. However, the small sample size, wide variability, and exploratory design indicate that these findings should be interpreted cautiously and require confirmation in larger adequately powered studies with adjusted analyses.

Regarding the mechanism underlying energy deposition within abdominal tissues, previous studies have proposed that multiple electromagnetic fields may interact within focal regions [17, 18].

Regarding the impact of EBD treatment on adipose tissue and fat reduction, previous studies have shown that temperatures within the fat layer may increase to approximately 45–55 °C at depths of 7–12 mm [19]. Such temperatures have been associated in experimental studies with biophysical changes in adipocytes and lipid release. Heating may selectively affect subcutaneous adipose tissue and has been associated in multiple studies with thermal effects linked to adipocyte apoptosis [20], without necessarily inducing a systemic inflammatory response detectable by C-reactive protein (CRP). However, other energy-based devices and treatment parameters have also been associated with adipocyte membrane disruption, inflammatory infiltration, and morphological changes consistent with early necrosis and adipocyte volume reduction. Recent evidence suggests that the adipocyte apoptotic index increases when fat temperature exceeds approximately 45 °C, while skin surface temperature remains considerably lower, indicating that thermal depth and dosimetry control may determine whether apoptosis predominates over necrosis and inflammation [21]. The observed decrease in leptin levels is consistent with the well-established relationship between adipose tissue mass and leptin secretion, as previous studies have shown that reductions in abdominal adipose tissue are associated with decreased leptin concentrations [22]. In the present study, intratissue temperature and adipocyte-level responses were not directly assessed; therefore, these mechanisms should be considered potential explanations for the MRI-observed reductions in abdominal adipose tissue rather than mechanisms directly demonstrated by this study.

This study represents a pilot investigation with a small sample size. Secondary biochemical analyses were exploratory, and no adjustment for multiple comparisons was applied; therefore, isolated statistically significant findings should be interpreted cautiously. Food intake in the EBD-only group and physical activity in both groups were not objectively monitored; therefore, the potential contribution of unmeasured lifestyle changes to the observed outcomes cannot be excluded. No additional follow-up assessments beyond the 3-month follow-up were performed; therefore, the durability of the observed changes beyond this period remains unknown. The findings should be considered exploratory and require confirmation in larger, adequately powered studies, including confidence intervals for mean differences, standardized effect sizes, and adjustment for variables that may influence treatment outcomes. Future studies should include direct assessment of basal metabolic rate and more precise dietary monitoring, including weighed food records, to quantify caloric intake and dietary adherence more accurately.

## Conclusion

In this preliminary study, the combined use of EBD and a semi-open hypocaloric diet with flexible food groups was associated with reductions in total, visceral, and subcutaneous abdominal fat in middle-aged adults of both sexes after 12 treatment sessions administered twice weekly over 6 weeks. Decreased leptin levels after treatment were consistent with fat reduction. At the 3-month follow-up, mean visceral fat remained below baseline in both groups. These findings describe preliminary reductions in abdominal adiposity in both treated groups but do not establish the independent effect of EBD because no untreated, sham, or diet-only comparator was included. However, larger adequately powered studies with standardized measurements are required to confirm these findings and assess safety and long-term outcomes.

The following abbreviations are used in this manuscript:

## Data Availability

No datasets were generated or analysed during the current study.

## Abbreviations

EBD: Energy-based device.
CRP: C-reactive protein.
SD: Standard deviation.
SF: Subcutaneous fat.
VAT: Visceral adipose tissue.
VF: Visceral fat.
MRI: Magnetic Resonance Imaging
